# Antiepileptic drugs and sexual dysfunction in patients with epilepsy

**DOI:** 10.1192/j.eurpsy.2025.2312

**Published:** 2025-08-26

**Authors:** S. Kolsi, A. Bouaziz, A. Haj Ali, K. Medhaffar, K. Zitoun, W. Abbes, E. Ellouz

**Affiliations:** 1 Psychiatry; 2 Neurology, Hospital University, Gabes, Tunisia

## Abstract

**Introduction:**

Epilepsy is a common disease that is mostly treated with antiepileptic drugs (AEDs). The sexual dysfunction (SD) side effects related to the use of AEDs have not received sufficient attention.

**Objectives:**

The aims of this study were to assess the prevalence of SD and to study the role played by the AEDs among patients with epilepsy.

**Methods:**

A cross-sectional and analytic study was conducted from September to December 2023, among patients with epilepsy follow up in the neurology outpatients of the University Hospital in Gabes (Tunisia), received AEDs, married for at least six months and sexually active. We collected the therapeutics data including type and number of prescribed AEDs and medication adherence, using pre-established form. SD was measured using the Arizona Sexual Experience Scale (ASEX) questionnaire.

**Results:**

Forty-five patients were enrolled (68.9% male and 31.1% female). The average age was 46.76 years (SD=12.39). The majority of patients had a low socio-economic status (64.4%). Carbamazepine and phenobarbital were the most commonly AEDs prescribed (57.8% and 53.3% respectively), especially as monotherapy (62.3%). Poor medication adherence was observed in 13 patients (28.9%). The frequency of SD among patients, based on ASEX questionnaire, was 44.4%. The factors associated with SD included carbamazepine and phenobarbital prescription (p=0.036 and p=0.045 respectively), double or multiple drug therapies (p=0.006) and poor medication adherence (p=0.033).

**Conclusions:**

SD is very common in patients with epilepsy. This seems to be related to AEDs such as using of carbamazepine and phenobarbital, polytherapy and poor medication adherence.

**Disclosure of Interest:**

None Declared

